# Cointegration and causality relationship of Indian stock market with selected world markets

**DOI:** 10.12688/f1000research.123849.3

**Published:** 2023-10-24

**Authors:** Farman Ali, Pradeep Suri, Tarunpreet Kaur, Deepa Bisht

**Affiliations:** 1 Department of Management, Uttaranchal University, Dehradun, Uttarakhand, 248001, India

**Keywords:** Stock market, Investors, Volatility, Global market, Unit root test, VECM, Johansen cointegration test

## Abstract

**Background:** The purpose of this study is to explore the trends and causes of established and emerging nations’ stock market integration with India. The National Stock Exchange (NSE) indices act as a counterweight to international market indices.

This study investigates the sustained interest of foreign investors in the Indian stock market in the wake of capital market reforms, as well as whether it moves in tandem with other markets in Asia and the United States.

**Methods:** Our study examined the possibility of cross-country cointegration between the largest economies and indices around the world using multiple financial econometric models, such as Augmented Dickey-Fuller, Unit Root, Correlation, and Johansen Cointegration.

**Results:** The findings of this study significantly support the notion that Indian and international financial markets are highly integrated. Vector error correction model indicates that the Indian market (NSE) is highly cointegrated with the US market (National Association of Securities Dealers Automated Quotations) and increased volatility signifies global contagion.

**Conclusion:** A cursory examination of the data reveals distinct investment and portfolio diversification options for global investors. This could assist regulators in formulating more effective rules regarding price discovery processes.

## 1. Introduction

Global stock market connections have been studied extensively. However, there has not been a great deal of academic interest in examining the interdependence of Emerging stock markets. An economic spillier occurs when one event sets off another event in a similar way, having an impact on economies both within and outside a country. In 2008, when Lehman Brothers collapsed in the U. S., the domino effect hit the economies worldwide including India ( Aloui *et al.*, 2016). Financial markets today are closely interconnected and driven by trust. Therefore, the developed and emerging markets have been a riveting field for the research of behavioural finance due to interlinked stock markets across the world. Lai *et al.* (2016) examined during the crisis, investors make errors of judgment as long as a group of investors takes irrational decisions that lead to worsening the situation of the stock market.

COVID-19 also delivered a shock to the economies of most countries, because of the nature of the interconnected global market ( Karkowska and Urjasz, 2021). The Indian stock market has been broadly resilient amidst the COVID-19 crisis so far, despite the devastating waves of the pandemic ( Kapoor *et al.*, 2020). However, the Indian economy has witnessed some impact from the global crash. Seth and Panda (2020) discussed that it is hard to pinpoint exactly the impact of any financial crisis on the Indian stock market, but it seems that the COVID-19 crisis has spilled over into some sectors. Numerous studies ( Shaikh, 2021; Dhall and Singh, 2020; Singh and Neog, 2020) have argued on insignificant causal linkage of the stock markets across the world. Some authors such as Zhang and Hamori (2021), have also focused on the possible factors and impacts of the global crisis on the Indian market. Hoque *et al.* (2007) investigated the co-movement of the Bangladesh market with those of Japan, the United States, and India and discovered evidence of a shared stochastic trend. In his paper, it was clear that shocks in the US affected Bangladesh, which is an emerging country. Rajwani and Mukherjee (2013) examined the interconnectedness of the Indian stock market with other Asian stock markets and discovered that the Indian stock market is not interconnected with any of the Asian stock markets, demonstrating the insensitivity of the Indian stock markets to other markets. Several studies have identified both short-term and long-term cointegration and interconnected financial markets between different economies of the world ( Stawiarski, 2021; Yarovaya and Lau, 2016). The global financial markets are closely interconnected and driven by the emotions of the investors ( Huang *et al.,* 2020). Hence, this manuscript focuses a causal linkage and cointegration between the global crisis and the Indian stock. In previous studies such as Mukherjee and Bose (2008) and Rajwani and Mukherjee (2013), Asian economies have been integrated with the economies of developed nations such as Japan and the United States.

In this study, we contribute to the literature on strategic financial decision-making for investors. Using vector auto regressive correlation (VAR) and vector error correction method (VECM) to assess the cointegration ( Yarovaya and Lau, 2016; Kanjilal and Ghosh, 2017; Aggarwal and Raja, 2019), have documented the multivariate cointegration-vector auto-regression method and their results indicate that the Indian stock market return depends on the world market returns. This study’s main objective is to comprehend how the volatility index of the Indian stock market might be impacted by the volatility index of some other countries. The Indian Capital Market has been examined by various academics at various times for its efficiency and co-integration. Utilizing the Engle-Granger test of co-integration, this study examines the co-integration characteristics of the Indian Capital Market with global markets in the post-liberalization period. Since there are so many markets and so much trade, no amount of research can adequately explain the behaviour. Indian capital markets and other capital markets around the world are investigated in this study.

Accordingly, the article is organized as follows: Section 2 summarizes existing studies that have used cointegration tests on the stock market. Section 3 describes the technique used in the article. Section 4 goes over the data, including possible structural breaks. Section 5 displays the cointegration test findings. Section 6 explores the ramifications of the findings, and Section 6 concludes.

## 2. Literature review

A series of recent studies ( Choudhary and Singhal, 2020; Kumar *et al.*, 2021; Sahoo and Kumar, 2021; Kartal *et al*., 2022) has investigated the cointegration relationship of the stock market amid crisis. Some studies ( He *et al*., 2020; Stawiarski, 2021) have explained the causal linkage of Indian stock market with Asian countries while in some other studies, a number of authors have recognized the comprehensive analysis by applying Generalized Autoregressive Conditional Heteroskedasticity Model ( Narasimha and Mushinada, 2020) and found very high volatility index of Indian stock market in comparison to developed countries during the COVID-19 crisis. While recent advances in the field of behavioural finance have highlighted the volatility clustering in the Indian stock market through the use of time series data analysis and predictive analytics. According to Khaing *et al.* (2020), stock trend extraction results matched genuine price movement. These patterns were retrieved with the news effect curve during the global recession 2008-2009. These criteria include timestamp conversion, identifying the necessary verb, stock reference, and identifying advanced trends. Chen (2021) created the new neural network model to develop the prediction model’s concepts. Belciug *et al.* (2021) evaluated the efficacy of a statistical learning framework using an algorithm based on a competitive/collaborative method for generating a reliable real-time forecast of the next stock market transaction price for a share during upswing of the market. Biswas *et al.* (2021) reviewed numerous models and approaches used in stock market prediction and focused on their advantages and disadvantages during the crisis. Numerous studies have argued that there is insignificant ( Goudarzi and Ramanarayanan, 2011) and significant ( Aggarwal and Raja, 2019) causal linkage among the stock markets across the world. Some scholars, such as Jain and Biswal (2016), have also focused on the possible drivers of rupee value decline and the influence of the 2014-2015 global crisis on the Indian stock market. It is well established that the global financial markets are closely interconnected Muthukumaran *et al.* (2011) and are driven by the emotions of investors. Several studies have identified interconnected financial markets between different economies of the world during1996-1997 crisis Yang *et al.* (2003) by applying VAR (vector auto-regression). There is a strong co-movement between developing and mature markets, and studies have found a larger correlation among well-established equities markets ( Bekaert and Harvey, 2003; Carrieri *et al.*, 2007). On the other hand, some early literature implies the existence of diversification benefits by investing in both developed and developing economies due to relatively lower correlations between stock markets of such economies ( Bekaert and Harvey, 1995; Foerster and Karolyi, 1999). Different experts have come to different conclusions about how stock markets around the world are related to each other. In addition, the relationship between global stock markets is temporally variable; national and global events can alter the nature and intensity of a relationship over time. Depending on its intensity, the phenomenon could last a short time or last for a very long time. Globalization has changed the relationship between various financial markets worldwide ( Can Inci *et al.*, 2011). Ahmad *et al.* (2005) have investigated only short term linkage whilst no significant linkage was found among the Indian Stock Market (NSE), Japanese (Nikkei) and US equity market (Dow Jones) amid crisis by using Granger-causality test. Menon *et al.* (2009) found that the Indian stock market did not have any causal linkage with the US and Japanese stock markets during the 2008 crisis period by hypothesizing Engle Granger test of co-integration. Siddiqui (2009) concluded that the Indian stock market is integrated with the global market. Some others studies such as Rajwani and Mukherjee (2013) have argued the Indian stock market is not interconnected with the Asian markets. Narayan *et al.* (2014) have investigated highly positive time varying bilateral correlations by analysing GARCH-dynamic conditional correlations.

Yarovaya and Lau (2016) have concluded the asymmetric causality linkage; their results indicating the coupling and decoupling of the Chinese stock market with the UK stock market. Nayak *et al*. (2016) applied machine learning algorithms and identified that the historical prices of the stocks are combined with sentiments of the investors. Nandy and Chattopadhyay (2019) have explained the unidirectional action from the world’s stock exchange indices over Indian stock market indices by carrying multivariate vector auto regression (VAR) analysis and Granger causality test. Aggarwal and Raja (2019) explored a long-run cointegration equation of the Asian and the Indian market by applying the Johansen cointegration model. Some other studies have also confirmed the bidirectional causality of Bombay Stock exchange indices with the US stock market indices by carrying Granger causality test ( Choudhary and Singhal, 2020). Seth and Panda (2020) have revealed the strong dynamic linkage between the global economies and Indian economy by analysing the Autoregressive (AR1), Generalized Auto Regressive Conditional Heteroskedasticity (GARCH 1,1) Model and Asymmetric Dynamic Conditional Correlation (ADCC) model to determine the coupling and decoupling. Apart from analysing only the degree of cointegration and contagion among the world market, several studies have hypothesized the global contagion on the volatility of Indian stock’s prices. Li *et al.* (2011) have confirmed that the GARCH model cannot completely account for all nonlinearity in simulated market amid global crisis. Goudarzi and Ramanarayanan (2011) have revealed the bilateral causality between the Bombay Stock Exchange and foreign institutional investors. Garg and Gulati (2013) examined the rationality of the Indian investors in the face of economic crisis and validated the use of rational pricing models. Ding *et al*. (2014) have indicated returns depend on the direction of the movement of buying and selling pattern and stock distinctiveness of individual holdings and on arbitrage constraint. Guyon (2014) suggested the models of path-dependent volatility provide excellent alternatives to the duopoly of local volatility and stochastic volatility that has dominated option pricing for the past twenty years.

The fractal structure has been studied by Mahalingam and Selvam (2014) with long term returns on the market. An adaptive multiplicative error model (MEM) with time-varying parameters was proposed by Härdle *et al.* (2015). A multiplicative error model (MEM) parameters are adaptively estimated through sequential testing. Bir *et al*. (2015) have investigated the highest volatility for open ended stocks in the Indian stock market. Kumari and Mahakud (2016) have explored the unidirectional causality between sentiment and stock market volatility. This study indicated that the market reacts more strongly to the impact or shock of negative or bearish mood than to positive or bullish sentiment. Bouri *et al.* (2017) have examined significant hypothesis about the existence of cointegration linkage and nonlinear volatilities of oil and gold in Indian stock market during the crisis. Abuzayed *et al*. (2018) demonstrated that the skewed Student-t FIGARCH (Fractionally integrated generalized autoregressive conditional Heteroscedasticity) model generates the most precise VAR forecast for a single day. Other studies have demonstrated the ripple effects of United States of America uncertainty on other developed markets, such as L. Fang *et al.* (2018). Boako and Alagidede (2018) have confirmed the correlation amongst African stock markets, regional and global markets by carrying a comprehensive analysis. Chuliá *et al*. (2018) have demonstrated that some crises have had a weak negative correlation with market synchrony. Wang *et al*. (2019) have confirmed that significant shocks have a substantial link with stock prices and assert that volatility is more susceptible to the asymmetric effect than extreme volatility. According to Xing and Yang (2019), firms that attract more individual investors provide higher returns with lower future stock price crash risk. It is widely accepted that a greater degree of correlation among stocks provides an early warning of the probability of crashes. It has been conclusively shown that herding is strongly evident during the fluctuations of market ( Shantha, 2019).

Some studies such as Fang *et al*. (2020); Wong *et al.* (2005); Daly (2003) also identified that the long-term volatility of the stock market depends upon many macroeconomic variables. While Lyócsa and Molnár (2020) discovered that the autoregressive coefficient was negative during COVID-19 (November 2019 to May 2020), they also discovered that uncertainty in the stock market and fear of viruses significantly influenced the breath of the autoregressive coefficient during the COVID-19 crisis. Narasimha and Mushinada (2020) point out that investors are concerned about cognitive biases and therefore adapt to changing market dynamics. ( Vo, 2020) studied the significant positive link between foreign investors and crash risk due to the asymmetry of information in the emerging market. Among BRICS (Brazil, Russia, India, China, and South Africa) a diverse responses to the stock market volatility reported including negative and positive shocks ( Salisu and Gupta, 2020). Cui and Zhang (2020) have suggested that negative information creates fear among the investor which leads to a larger stock price crash risk. According to Nikkinen and Peltomäki (2020), investors’ crash worries are examined by using published newspaper articles and web search volumes. They examine how information supply and demand relate to investor anxiety and their effects on stock market returns, implying that media contribute to the efficiency of information transmission. He *et al.* (2020) have examined the futures markets ability to price discover by margin trading on the stock market. Kumar and Misra (2020) have found widespread evidence of long-term similarity among the NIFTY 50 index and the global market. Naik *et al*. (2020) used GARCH (Generalized Auto-Regressive Conditional Heteroskedasticity) to calculate stock return error distribution. When time series data are heteroskedastic and volatile, the GARCH model is best. GARCH effectively predicted stock market crises (1995-2019) using State Bank of India and Infosys datasets. Elyasiani *et al*. (2021) analysed market greed by including the skewness index, which measures investor enthusiasm as opposed to investor fear. Previous studies have mainly exclusively explored the integration of Asian economies with other developed nations such as the United States and Japan during the period from 3 ^rd^ January 2011 to 29 ^th^ December 2017 in the midst of a global financial crisis. According to the literature, during the COVID-19 crisis, domino effects had both short-term and long-term negative consequences on economies all around the world ( Salisu and Gupta, 2020).

However, there is a contradiction in the argument made by Aggarwal and Raja (2019); Rajwani and Mukherjee (2013) and Menon *et al.* (2009). Long-term cointegration of stock markets was not discovered, but correlation analysis revealed that stock market integration was growing with time.

Even though the majority of the COVID-19 economic crisis has passed, further research is still needed on the developed and rising Asian markets. In addition, it would be interesting to know whether the effect of the crisis on the Indian stock market persists over time and, if so, for how long. Do the recent shocks to global markets alter the standard deviation of forecasting errors in the Indian stock market? Contributing to existing theory and strategic financial decision-making for investors, this paper offers valuable insights. In particular, the authors explore how the global volatility index’s shock influences the Indian volatility index, how long the impact lasts, and the degree and sign of the effect.

## 3. Methods

In our study, we examined data from several major indices, including the NSE in India, the NIKKEI (Japan’s Nikkei 225 Stock Average) in Japan, NASDAQ (National Association of Securities Dealers Automated Quotations), DJI (Dow Jones Industrial Average) and S&P (Standard and Poor index) in the United States, the FTSE (Financial Times Stock Exchange) in the UK, the DAX (Deutscher Aktien Index) in Germany, the FTXIN (FTSE–Xinhua China A50 Index) in China, Cotation Assistée en Continu (CAC) benchmark of France stock market and the Hang Seng in Hong Kong. The descriptive statistics for all indexes’ returns are shown in Table 3. We compiled and collected the data from different websites (including Yahoo, Investing.com and NSE India) over the long-term, encompassing a significant portion of the recession from January 1, 2008, to December 2, 2021. The time period covered by the research has been selected to provide an in-depth look at the worldwide correlations that have an effect on the Indian stock market over the long term. Although the trading hours of each stock exchange varies, the time frame is the exact same for all indexes. Therefore, in order to examine the group statistics, we have taken the common sample. We calculated the daily return by applying the [Return=log (Closing price of indices/Closing price of indices (-1))] equation over the closing price. We analysed the data using the EViews 12 (University Version) software package. The Johansen cointegration test ( Menon *et al*., 2009) ^1^ is used to demonstrate a long-term link between variables. To determine short-term and long-term associations between variables, the vector error correction model was utilised followed by Ezeibekwe (2021).

### Hypothesis

*H0: There is no long-term linear interdependency between the NSE index and the global index.*

*H1: There is a long-term linear interdependency between the NSE index and the global index.*

### Vector error correction model

This model is adopted when variables are cointegrated. VAR indicates the cointegration of the variables over the short term. In addition, by incorporating error correction terms, the model examines the long-term causal relationship ( Golder *et al.*, 2020). The number of variables can be measured by using the equations below. The vector autoregressive model of order 1 is called VAR (1):

$$x_{t,1}=\alpha_{1}+\phi_{11}x_{t-1,1}+\phi_{12}x_{t-1,2}+\phi_{13}x_{t-1,3}+w_{t,1}$$ (1)

$$x_{t,2}=\alpha_{2}+\phi_{21}x_{t-1,1}+\phi_{22}x_{t-1,2}+\phi_{23}x_{t-1,3}+w_{t,2}\;$$ (2)

$$x_{t,3}=\alpha_{3}+\phi_{31}x_{t-1,1}+\phi_{32}x_{t-1,2}+\phi_{33}x_{t-1,3}+w_{t,3}$$ (3)

Engle-Granger model is used to measure ( Stawiarski, 2021) cointegration between the indices from the NSE and global indices such as Japan (NIKKEI), U.S. (NASDAQ, DJI, and S&P), UK (FTSE), Germany (DAX), China (FTXIN), Hong Kong (HANG SENG) and France (CAC). In 1981, Granger introduced the concept of cointegrated multivariate time series to demonstrate linear combinations of stationary variables imply that there is a long-term relationship ( Engle *et al.*, 1987).

### Unit root test

A regression model must have stationary conditions to prevent spurious regressions. Our study examines stationary conditions by using the ADF test (Augmented Dickey-Fuller) ( Cheng *et al.,* 2021). The null hypothesis is rejected if the ADF value (calculated) is less than the critical levels (1%, 5%, and 10%). The cointegration test follows the unit root test for measuring the extent of co-movement of long-term relationships among indices ( Cheng *et al.,* 2021).

### Johansen cointegration test

In Johansen cointegration ^2^, the number of independent linear combinations (k) that give a stationary process is determined for (m) time series variables. The results are given as cointegration ranks. There is no cointegration relationship when the rank is 0, and there is a cointegration equation when the rank is 1, and so on. Johansen cointegration test analyses the relationship between the NSE index and global index by using Eigen-values and trace statistics. Integration is based on how many times a series need to be differentiated to produce a stationary series. The Johansen cointegration test is a statistical method used to determine the presence of cointegration among a set of time series variables. Cointegration implies a long-term relationship between these variables, which is essential for various econometric models and time series analysis. In the test, ‘P’ denotes the number of cointegration vectors (also known as the cointegration rank). It represents the maximum number of linearly independent combinations of the variables that exhibit a stationary behaviour. ‘m’ represents the number of variables in the system being tested, and ‘k’ is the number of deterministic terms (e.g., intercept or time trend) included in the model. The first difference generates an integrated series known as I (1). As a result, a time series with I (0) is stationary; if I (1), the level is stationary and the change is stationary. The equation below determines cointegration:

P=m−k

Null Hypothesis; H0: When k = 0, then p = m, there is no linkage among the variables ^3^.

Alternate Hypothesis; H1: 0 < k < m, 0 < p < m There is a significant linkage among the variables.

## 4. Descriptive statistical analysis

**Figure 1.**
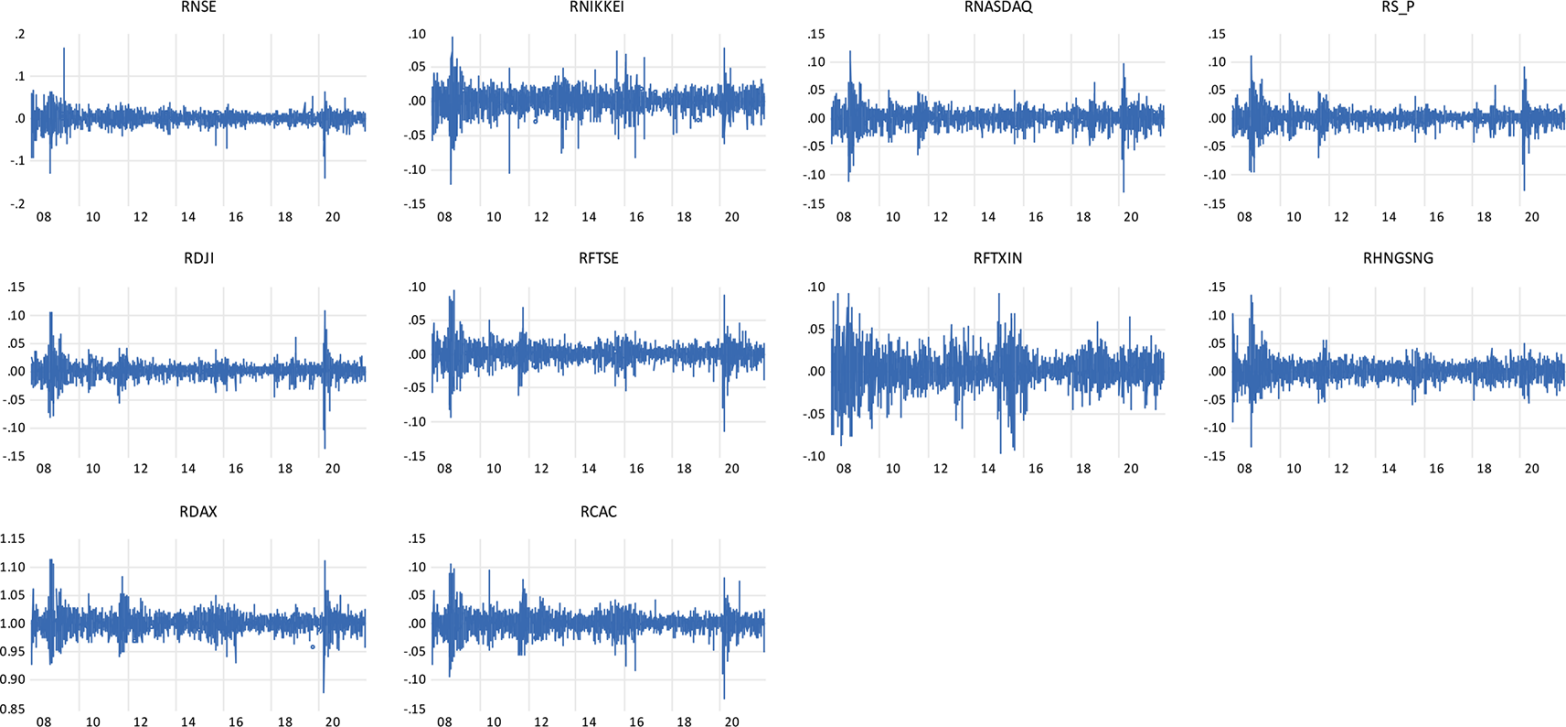
Volatility clustering plot of daily returns to NSE, NIKKEI, NASDAQ, S&P, DJI, FTSE, FTXIN, HANG SENG, DAX AND CAC INDICES (Source: author’s calculations).

**Figure 2.**
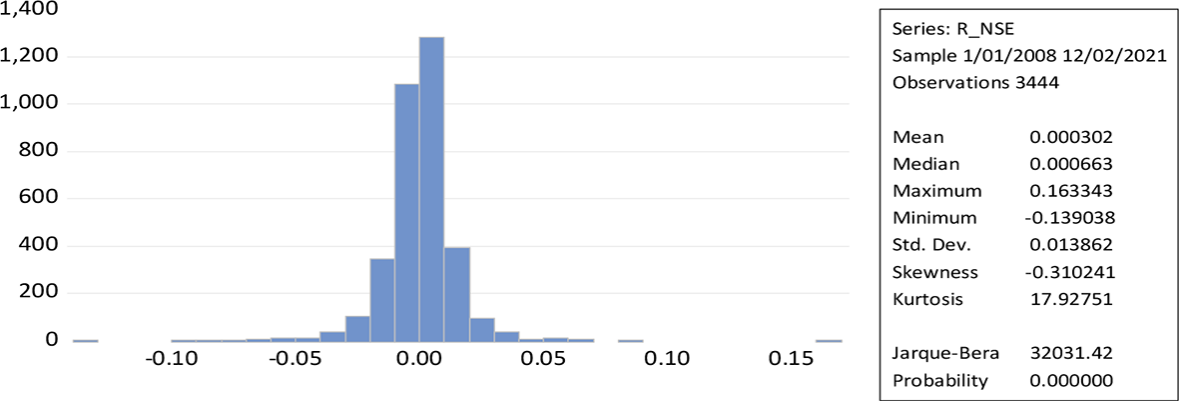
Time series plot of returns to NSE (Source: author’s calculations).

Individual observations show large shifts during crisis times are further followed by large shifts in the return of the NSE indices and the global indices representing the wild and calm periods of volatility clustering. Figure 1 shows the clustering of the volatility for the daily return of indices, firstly noted by Mandelbrot and Taylor (1967) ‘large changes tend to be followed by large changes, of either sign, and small changes tend to be followed by small changes.’ Figure 2 represent the leptokurtic statistical distributions with kurtosis greater than three results to a greater extent of volatility because of positive or negative shocks in the stock market. While the Jerque-Bera of NSE daily return (32031.42) measures the high volatility. The shape of the curve and the value of kurtosis along with the low probability value reveal the possibility for the rejection of the null hypothesis. The skewness measures the asymmetry of a time series over a given period (normal skewness; 0, positive skewness; long right tail, negative skewness; long left tail).

Table 1 indicates that the left-hand tail of the German Blue chip stock market (DAX) is smaller than the right-hand tail. In other indices, a negative skewness (the left-hand tail is larger than the right-hand tail) indicates a scenario of small wins and few large losses for investors. Table 1 provides descriptive statistics on daily returns, highlighting Jarque-Bera standard deviation using the same sample for all indices. The NSE returns over the entire period sample shows a negatively skewed distribution of the sample (Jaque-Bera 33817.66, standard deviation 0.013768, Kurtosis 20.31397). In Japan, the statistical moments of the NIKKEI were Jarque-Bera 5525.19, standard deviation 0.0151, and Kurtosis 10.171). Jarque-Bera values for US Market (NASDAQ; 10447.74, standard deviation 0.014376, and Kurtosis 17.26778), (S&P; 23085.57, standard deviation 0.013164), and (DJI; 33669.34, standard deviation 0.012713, and Kurtosis 20.25097). These all show a similar leptokurtic distribution with negative skewness.

**Table 1.** Descriptive statistics (common sample) for daily returns of indices.

Indices	RNSE	NIKKEI	RNASDAQ	RS_P	RDJI	RDAX	RCAC	RFTSE	RFTXIN	RHNGSNG
Mean	0.0003	0.0000	0.0004	0.0002	0.0002	1.0003	0.0001	0.0000	-0.0001	-0.0002
Median	0.0005	0.0005	0.0011	0.0007	0.0006	1.0007	0.0005	0.0004	-0.0001	0.0003
Maximum	0.1633	0.0773	0.1185	0.1096	0.1076	1.1140	0.1059	0.0938	0.0920	0.1341
Minimum	-0.1390	-0.1211	-0.1300	-0.1277	-0.1384	0.8776	-0.1310	-0.1151	-0.0942	-0.1358
Std. Dev.	0.0138	0.0151	0.0144	0.0132	0.0127	0.0143	0.0147	0.0123	0.0167	0.0146
Skewness	-0.2609	-0.8050	-0.3772	-0.5604	-0.5324	0.0784	-0.1622	-0.2589	-0.1468	-0.0441
Kurtosis	20.3140	10.1740	12.5983	17.2678	20.2510	12.3473	11.7053	13.8112	7.2921	13.1632
Jarque-Bera	33817.6600	5525.1930	10447.7400	23085.5700	33669.3400	9850.2390	8553.0460	13203.8800	2086.0790	11642.6900
Probability	0.0000	0.0000	0.0000	0.0000	0.0000	0.0000	0.0000	0.0000	0.0000	0.0000
Sum	0.7811	-0.0068	1.2053	0.6292	0.5666	2705.7210	0.1920	-0.0574	-0.3805	-0.5348
Sum Sq. Dev.	0.5126	0.5597	0.5588	0.4686	0.4370	0.5557	0.5822	0.4058	0.7521	0.5733
Observations	2705.0000	2705.0000	2705.0000	2705.0000	2705.0000	2705.0000	2705.0000	2705.0000	2705.0000	2705.0000

Source: author’s calculations.

It is evident from the descriptive statistics that the significant value probabilities and the Jerque-Bera calculation indicate that the residual distribution of daily returns, like other indices (FTSE) Jarque-Bera 13203.88, standard deviation 0.012250 with Kurtosis value 13.81124), Germany (DAX) Jarque-Bera 9850.239, standard deviation 0.014335 with Kurtosis value 12.34726), Hong Kong (HANG SENG) Jarque-Bera 11642.69, standard deviation 0.0126 and Kurtosis 19.89 and France (CAC) Jarque-Bera 8553.046. The values of skewness’ and kurtosis observed the volatility clustering and specify the distribution of all indices (variables) is leptokurtic. Only Germany (DAX) is positively skewed, which specify that the huge gain covers the small losses of investors. The Indian stock market exhibits a positive correlation with the world market, as seen in Table 2; however, the correlation of all variables is not strong enough to explain the cointegration.

**Table 2.** Correlation matrix for the daily returns of indices.

Dependent variable	RNSE	RNIKKEI	RNASDAQ	RFTXIN	RDAX	RHNGSNG	RFTSE	RDJI	RCAC	RS_P
RNSE	1.000	0.384	0.259	0.287	0.426	0.567	0.441	0.324	0.423	0.322
RNIKKEI	0.384	1.000	0.141	0.330	0.345	0.600	0.373	0.188	0.366	0.181
RNASDAQ	0.259	0.141	1.000	0.113	0.571	0.238	0.519	0.876	0.544	0.931
RFTXIN	0.287	0.330	0.113	1.000	0.183	0.563	0.208	0.110	0.193	0.116
RDAX	0.426	0.345	0.571	0.183	1.000	0.407	0.848	0.646	0.921	0.643
RHNGSNG	0.567	0.600	0.238	0.563	0.407	1.000	0.436	0.271	0.402	0.273
RFTSE	0.441	0.373	0.519	0.208	0.848	0.436	1.000	0.612	0.894	0.606
RDJI	0.324	0.188	0.876	0.110	0.646	0.271	0.612	1.000	0.627	0.976
RCAC	0.423	0.366	0.544	0.193	0.921	0.402	0.894	0.627	1.000	0.624
RS_P	0.322	0.181	0.931	0.116	0.643	0.273	0.606	0.976	0.624	1.000

Source: author’s calculations.

**Figure 3.**
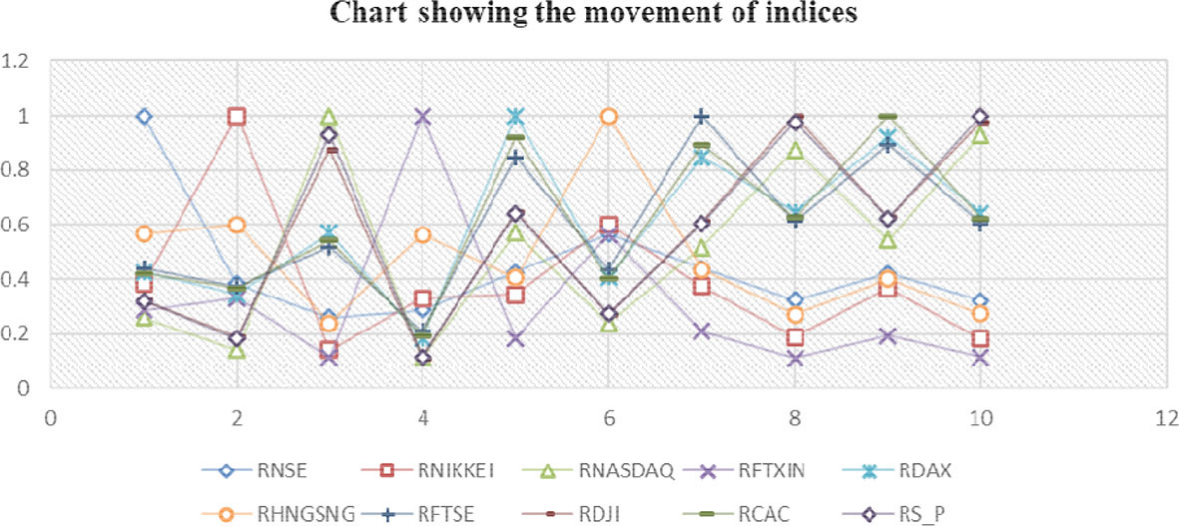
Movement of indices.

Table 2 examines the correlation trends between the Indian market and a few selected global markets, indicating that there is a relationship between the Indian stock market and other markets; nevertheless, Figure 3 illustrates that this connection is sometimes parallel to the global economy.

It is said that the “flap of a butterfly’s wings in Brazil could set off a tornado in Texas” ( Lorenz, 2000) that is not true in the Indian context. The Indian stock market is still linked to the world market because the NSE has a positive correlation with all global indexes, despite the fact that this correlation has been reducing significantly.

### Unit root test analysis

A unit root test determines whether a time series is stationary. The null hypothesis defines time series as having a unit root, while the alternative hypothesis defines them as being stationary. Table 3 indicates the value of t statistics in all variables exceeds the critical value.

**Table 3.** Augmented dickey-fuller test statistic.

Null Hypothesis: D (R indices) has a unit root				
Exogenous: Constant				
Lag Length: 2 (Automatic - based on AIC, max.lag=2)				
Augmented Dickey-Fuller test statistic			t-Statistic	Prob. *
		D (RNSE)	-49.54543	0.0001
		D (RNIKKEI)	-49.86084	0.0001
		D (NASDAQ)	-55.14356	0.0002
		D (RHNGSNG)	-57.60954	0.0031
		D (RFTXIN)	-56.95976	0.0001
		D (RFTSE)	-61.81385	0.0001
		D (RDJI)	-54.49088	0.0001
		D (RDAX)	-59.06823	0.0001
		D (RCAC)	-61.97524	0.0001
		D (RS_P)	-54.97958	0.0001
Test critical values:	1% level	-3.432586		
	5% level	-2.862414		
	10% level	-2.567280		

Source: author’s calculations.

* MacKinnon (1996) one-sided p-values.

In this case, these variables were tested again at a difference of one, and the result indicated a null hypothesis, i.e., that the series of all variables are integrated over four lag of order one. To perform Johansen’s cointegration test we employed the VAR lag order selection criterion. Table 4 identifies the AIC value at the third lag so we examined the cointegration test by using the third lag, recommended by AIC. A Japanese statistician ( Akaike, 1974) developed the Akaike information criterion. It currently serves as a paradigm for the foundations of statistics and is also commonly employed for statistical inference. The Akaike information criterion (AIC) is an estimator of prediction error and, therefore, relative model quality for a given set of data. Given a set of data models, AIC determines the quality of each model in comparison to the other models. When a statistical model is employed to depict the process that generated the data, the representation is virtually never perfect; as a result, some information is lost. AIC assesses the relative amount of information lost by a particular model; the less information a model loses, the higher the model’s quality.

**Table 4.** VAR Lag order selection criteria.

Endogenous variables: RS_P RNSE RNIKKEI RNASDAQ RHNGSNG RFTXIN RFTSE RDJI RDAX RCAC						
Exogenous variables: C						
Lag	Log L	LR	FPE	AIC	SC	HQ
0	21099.53	NA	1.35e-42	-68.03073	-67.95928	-68.00295
1	21497.50	781.8252	5.16e-43	-68.99193	-68.20601 *	-68.68644 *
2	21620.40	237.4761	4.79e-43	-69.06580	-67.56542	-68.48259
3	21721.71	192.4845	4.77e-43 *	-69.07002 *	-66.85516	-68.20909
4	21802.79	151.4382	5.08e-43	-69.00899	-66.07966	-67.87034
5	21873.51	129.8028	5.59e-43	-68.91454	-65.27074	-67.49817
6	21977.24	187.0576	5.54e-43	-68.92659	-64.56831	-67.23250
7	22079.51	181.1104	5.52e-43	-68.93390	-63.86115	-66.96209
8	22171.27	159.5397 *	5.70e-43	-68.90731	-63.12009	-66.65778

Source: author’s calculations.

LR: sequential; modified LR test statistic, FPE: Final prediction error; AIC: Akaike information criterion; SC: Schwarz information criterion; HQ: Hannan-Quinn information criterion.

* Indicates lag order selected by the criterion (each test at 5% level).

## 5. Results

### Johansen’s cointegration test results

Table 5 shows the statistical values of Johansen’s cointegration test. In addition, it provides information about the maximum Eigen-value. Statistics for all variables dependent (NSE) and independent (World’s indices) demonstrate a perfect correlation of cointegration. Since the calculated value of statistics and maximum Eigen-value are greater than the critical value (at 5% level) of ( MacKinnon *et al.,* 1999), the null hypothesis of no cointegration is rejected in favour of the alternative hypothesis of cointegration. Therefore, the Indian stock market and the global stock market have ten cointegration equations.

**Table 5.** Johansen’s cointegration test.

Lags interval (in first differences): 1 to 3				
Unrestricted cointegration rank test (Trace)				
Hypothesized No. of CE(s)	Eigen-value	Trace statistic	0.05 Critical value	Prob. **
None *	0.334300	3261.797	239.2354	0.0000
At most 1 *	0.285750	2753.151	197.3709	0.0000
At most 2 *	0.268835	2332.498	159.5297	0.0000
At most 3 *	0.231890	1941.102	125.6154	0.0000
At most 4 *	0.222216	1611.324	95.75366	0.0000
At most 5 *	0.212214	1297.192	69.81889	0.0000
At most 6 *	0.206747	999.0299	47.85613	0.0000
At most 7 *	0.190017	709.5135	29.79707	0.0000
At most 8 *	0.170580	446.0860	15.49471	0.0000
At most 9 *	0.156201	212.3006	3.841465	0.0000
Trace test indicates 10 cointegrating eqn (s) at the 0.05 level.				

Hypothesized No. of CE(s)	Eigen value	Max-Eigen Statistic	0.05 Critical value	Prob. **
None *	0.334300	508.6457	64.50472	0.0000
At most 1 *	0.285750	420.6532	58.43354	0.0000
At most 2 *	0.268835	391.3954	52.36261	0.0000
At most 3 *	0.231890	329.7780	46.23142	0.0000
At most 4 *	0.222216	314.1328	40.07757	0.0000
At most 5 *	0.212214	298.1618	33.87687	0.0000
At most 6 *	0.206747	289.5164	27.58434	0.0000
At most 7 *	0.190017	263.4275	21.13162	0.0000
At most 8 *	0.170580	233.7855	14.26460	0.0000
At most 9 *	0.156201	212.3006	3.841465	0.0000

Max-eigenvalue test indicates 10 cointegrating eqn (s) at the 0.05 level.

Source: author’s calculation.

* Denotes rejection of the hypothesis at the 0.05 level.

** MacKinnon *et al.* (1999) p-values.

We employ VECM to evaluate the short-run characteristics of the cointegrated variables in order to ascertain whether they remain stationary over time ( Table 6). If cointegration has been found between series, we are aware that there is a long-term equilibrium link between them.

**Table 6.** Vector error correction estimates.

Cointegrating Eq:	CointEq1	CointEq2	CointEq3
NSE (-1)	1.000000	0.000000	0.000000
NIKKEI (-1)	0.000000	1.000000	0.000000
NASDAQ (-1)	0.000000	0.000000	1.000000
SP (-1)	-5.908983	0.686667	-7.595660
	(0.99997) [-5.90918]	(3.23212) [ 0.21245]	(0.39953) [-19.0114]
DJI (-1)	0.189825	-1.192373	0.215697
	(0.11843) [ 1.60291]	(0.38278) [-3.11505]	(0.04732) [ 4.55862]
DAX (-1)	0.632029	-2.710561	-0.179478
	(0.16343) [ 3.86730]	(0.52824) [-5.13131]	(0.06530) [-2.74863]
CAC (-1)	-0.460907	10.90881	2.086912
	(0.38206) [-1.20637]	(1.23490) [ 8.83374]	(0.15265) [ 13.6712]
FTSE (-1)	-1.492381	-5.607010	-0.224850
	(0.35493) [-4.20473]	(1.14721) [-4.88751]	(0.14181) [-1.58557]
XIN (-1)	-0.053049	-0.524904	0.053855
	(0.09164) [-0.57891]	(0.29619) [-1.77219]	(0.03661) [ 1.47092]
HNGSNG (-1)	0.023054	0.574167	-0.058868
	(0.05353) [ 0.43068]	(0.17302) [ 3.31852]	(0.02139) [-2.75245]
C	6557.587	12741.45	2171.077

The effectiveness of market connectivity is measured by residual diagnostics for heteroscedasticity, which aid in identifying heteroscedasticity ( Table 7). One can determine whether the model can be trusted based on this. Probability scores over 0.05 indicate that the model should work for the Indian stock market.

**Table 7.** VEC residual heteroskedasticity tests (levels and squares) joint test.

Chi-sq	df	Prob.
8093.609	1430	0.2731

The Jarque-Bera test in statistics determines if sample data have skewness and kurtosis that are consistent with a normal distribution. The result of Table 8 shows that the regression residual is normally distributed since the p value is greater than 0.05.

**Table 8.** VEC residual normality tests: null hypothesis: residuals are multivariate normal.

Component	Skewness	Chi-sq	df	Prob. *
1	-0.030822	0.234808	1	0.6280
2	0.341632	28.84747	1	0.2776
3	0.627158	97.21750	1	0.1832
4	-0.117999	3.441499	1	0.8308
5	0.381231	35.92239	1	0.6795
6	-0.287373	20.41181	1	0.5404
7	1.312546	425.8131	1	0.2081
8	-0.070094	1.214358	1	0.2705
9	-0.003174	0.002490	1	0.9602
10	-0.355615	31.25722	1	0.0000
Joint		644.3626	10	0.2271

Component	Kurtosis	Chi-sq	df	Prob.
1	20.83695	19659.44	1	0.849
2	10.34838	3336.671	1	0.186
3	12.81838	5956.751	1	0.273
4	5.912879	524.2937	1	0.251
5	6.440253	731.3253	1	0.275
6	10.60865	3577.214	1	0.220
7	89.73248	464829.3	1	0.307
8	4.173781	85.13416	1	0.401
9	4.787424	197.4173	1	0.285
10	10.62113	3588.961	1	0.849
Joint		502486.5	10	0.643

Component	Jarque-Bera	df	Prob.
1	19659.68	2	0.279
2	3365.519	2	0.252
3	6053.969	2	0.276
4	527.7352	2	0.318
5	767.2477	2	0.355
6	3597.626	2	0.403
7	465255.1	2	0.279
8	86.34852	2	0.252
9	197.4198	2	0.357
10	3620.218	2	0.348
Joint	503130.8	20	0.294

* Approximate p-values do not account for coefficient.

The null hypothesis is accepted for the sample based on the serial correlation test results, which show that the p-value is greater than 0.05 at the 5% significance level. The serial correlation verifies the accuracy and validity of the results of the cointegration test ( Table 9).

**Table 9.** Serial correlation test.

Null hypothesis: No serial correlation at lag h						
Lag	LRE * stat	df	Prob.	Rao F-stat	df	Prob.
1	1143.845	100	0.0000	12.03171	(100, 1039...	0.3803
2	1678.684	100	0.0000	18.12598	(100, 1039...	0.3741

Null hypothesis: No serial correlation at lags 1 to h						
Lag	LRE * stat	df	Prob.	Rao F-stat	df	Prob.
1	1143.845	100	0.0000	12.03171	(100, 1039...	0.2775
2	2011.990	200	0.0000	10.79347	(200, 1296...	0.1831

* Edgeworth expansion corrected likelihood ratio statistic.

**Figure 4.**
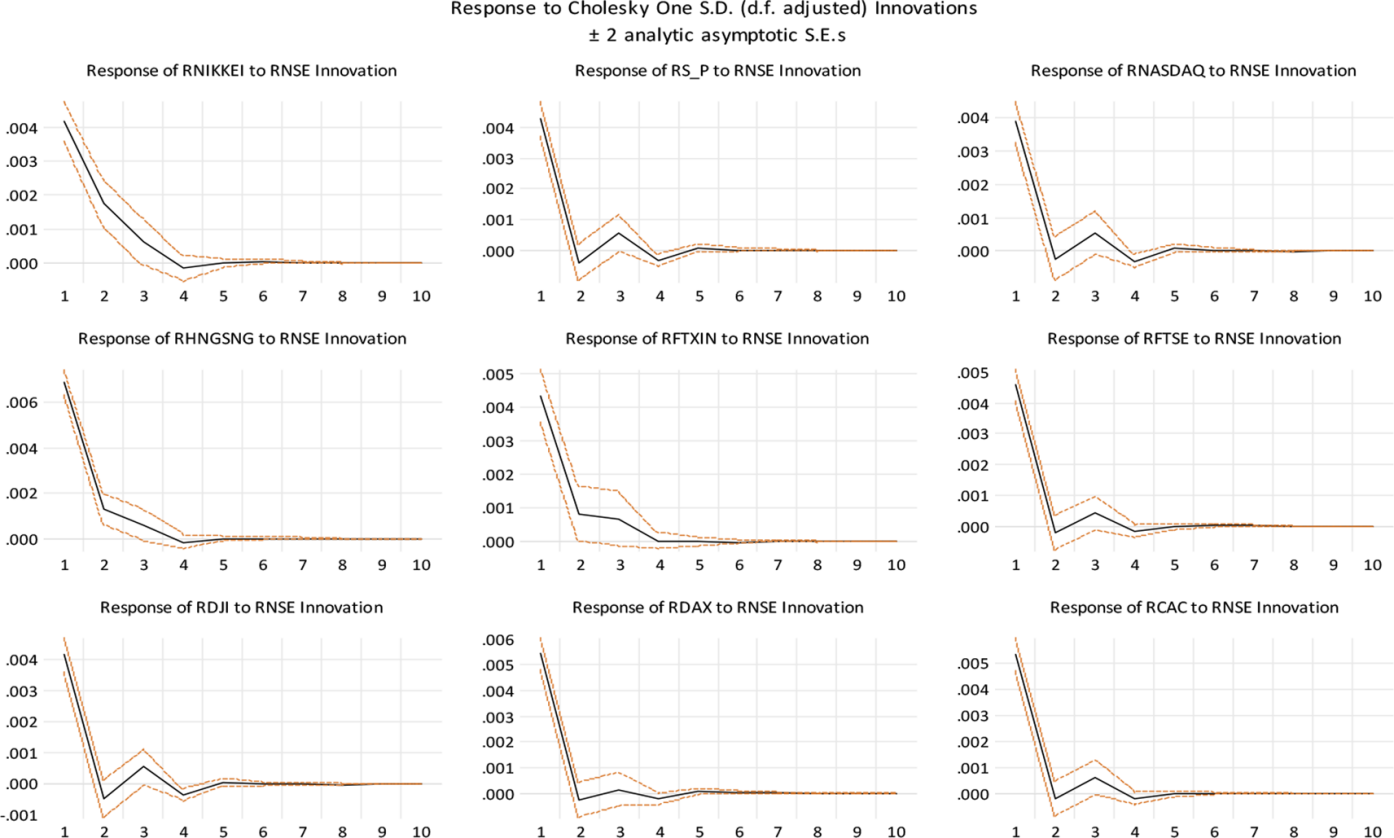
Impulse response functions (Source: authors calculations).

In Figure 4, we show impulse response functions associated with the non-factorized one standard deviation of innovations for the Indian stock market and the stock markets of some of India’s top trading partners and developed nations. It depicts the negative impulse responses of the Chinese and Japanese stock markets. Interestingly, both markets are responding similarly to a shock to the Indian stock market in their respective stock markets. Geographic proximity may be the reason for this. The established stock markets of the United States and other economies have a distinct trend. It demonstrates that these markets are in fact developed and have a robust positive impulse reaction to the Indian stock market.

### Empirical results

This study shows innovations in the Indian stock market do indeed propagate to India’s top trading partner’s stock markets in a time-varying manner. In this study, the U.S. stock market appears to be the most influential. The analysis of the ADF statistics ( Table 3) confirmed the unit root series for all indices and then we determined lag order using the VAR Lag Order Selection Criteria ( Table 4) to carry out causality test. To perform the cointegration test, we selected the AIC, indicating the third lag. Table 5 reveals the critical value is less than trace statistics and Max-Eigen values, which rejects the null hypothesis of no causal links, resulting in cointegration equations among all variables. Panel C of Table 10 predicts the long-term cointegration equation for the Indian stock market, which suggests that Japan (NIKKEI), US (NASDAQ, DJI, UK (FTSE), Germany (DAX), Hong Kong (HANG SENG) and France (CAC) indices have a positive correlation with Indian Indices (NSE), while China (RFTXIN) and US (S&P) indices have a negative correlation, considering ceteris paribus on Indian stock market. As Song *et al*. (2021) have documented supporting evidence, our findings are consistent with the idea that global financial crises have positively influenced interdependence of stock markets in Asian countries. Based on the coefficients, the linkage between the Indian stock market and the global market is statistically significant at a 1% level. Additionally, the Chinese stock market indices and S&P show a negative impact on Indian stock markets and the NASDAQ and Dow Jones indices in the US, Hong Kong (HANG SENG), and Japan (NIKKEI) have shown the strongest long-term correlation with Indian markets. The impulse reaction of NIKKEI to unit shock in NSE causes a little decrease in NSE on days 3 and 4, but an increase on day 6. Between days 8 and 10, this effect increases marginally. Similarly, when a unit shock is applied to NSE, NIKKEI displays a mixed reaction. This is evidence that the Japanese and Indian stock markets are not fully integrated. The impulse response of S&P to a unit shock on the NSE demonstrates that it has a negative impact on the SENSEX on the second day and a positive impact on the third day. It indicates that the NSE is highly cointegrated with the S&P and in the same way, DJI, NASDAQ, FTSE, and CAC. In addition, HNGSNG, DAX, and FTXIN do not have a significant impact on the Indian stock market. Similar findings are reported by Choudhary and Singhal (2020) and Mukherjee and Bose (2008). In addition, we find that our findings are in line with those of Tripathi and Sethi (2012), Hoque *et al*. (2007), etc. The correlation results are consistent with Seth and Panda (2020) indicated that the Indian stock market index has a strong positive correlation with the US stock market index.

**Table 10.** Unrestricted cointegrating coefficients (normalized by B'*S11*B=I).

Unrestricted Cointegrating Coefficients (normalized by b'*S11*b=I): PANEL A										
RNSE	RNIKKEI	RNASDAQ	RDAX	RFTXIN	RHNGSNG	RFTSE	RDJI	RCAC	RS_P	
48.80460	-43.88065	-55.52894	-21.15840	12.51878	-73.39477	-35.78659	-267.9007	-121.6060	598.1070	
-5.517466	-143.6456	-20.65795	-13.84757	25.57719	-63.85301	-56.58934	162.7164	203.5676	-83.88764	
-39.61267	38.78913	-261.1437	34.75907	-59.52097	81.86909	-163.6048	11.16332	38.26515	360.8356	
-115.2163	-23.96577	211.9508	159.3792	-16.68902	88.49715	-134.9213	-39.30057	-48.41080	-124.4930	
-81.29088	40.92984	114.9585	-253.6834	-2.066002	9.699304	45.33348	458.7454	93.36657	-424.4553	
80.23515	23.15905	43.81374	-48.05538	-22.90149	-37.32344	-302.8736	88.22049	185.3251	-145.3880	
24.65592	64.02229	22.36051	-104.8731	92.24334	-65.68420	-141.4215	-80.91578	195.7031	93.04734	
2.437710	-15.75192	106.6659	-220.3579	-77.29343	49.72520	40.41041	-303.8509	246.6365	163.9945	
86.06294	-54.75510	240.5182	-8.903653	-27.85157	92.37009	9.030500	499.7341	-46.76613	-668.2037	
25.49272	117.3313	163.7942	119.9363	-15.46875	-173.0843	85.64101	150.4572	-113.9755	-297.0598	

Unrestricted Adjustment Coefficients (alpha): PANEL B										
**D (RNSE)**	0.000223	0.001920	-0.000243	0.003770	0.003266	-0.000131	-0.002115	-0.000944	-0.003319	0.000616
**D (RNIKKEI)**	0.003035	0.004928	-0.002069	0.001040	-0.001143	0.000154	-0.003472	-0.000489	-1.93E-05	-0.001033
**D (RNASDAQ)**	-0.002523	0.000326	-0.001496	-0.002115	-0.000311	0.002292	-0.003464	-0.002115	-0.002876	-0.001172
**D (RDAX)**	0.002884	-0.001214	-0.002879	-0.001209	0.002868	0.002775	-0.002972	-0.000614	-0.002005	-0.001536
**D (RFTXIN)**	0.000967	0.000476	0.003153	0.000814	-9.52E-05	0.002660	-0.005898	0.003115	-4.87E-05	0.002692
**D (RHNGSNG)**	0.003160	0.002852	-0.001137	-0.000175	0.000507	0.001859	-0.002674	-0.000510	-0.002274	0.003158
**D (RFTSE)**	0.002459	-0.000696	-0.000989	0.000880	0.000816	0.003947	-0.001745	-0.001658	-0.001533	-0.001124
**D (RDJI)**	-0.001413	-0.000840	-0.003599	-0.000205	-0.000701	0.002373	-0.002824	-0.000719	-0.002505	-0.000768
**D (RCAC)**	0.003391	-0.002547	-0.002221	-5.26E-05	0.001494	0.002433	-0.003637	-0.002292	-0.001565	-0.001232
**D (RS_P)**	-0.002145	-0.000331	-0.003237	-0.000622	-0.000140	0.002644	-0.003156	-0.001440	-0.002185	-0.000699

1 Cointegrating Equation(s):		Log likelihood	41945.85		PANEL C				
Normalized cointegrating coefficients (standard error in parentheses)									
RNSE	RNIKKEI	RNASDAQ	RDAX	RFTXIN	RHNGSNG	RFTSE	RDJI	RCAC	RS_P
1.000000	-0.899109	-1.137781	-0.433533	0.256508	-1.503849	-0.733263	-5.489252	-2.491691	12.25514
	(0.18129)	(0.39565)	(0.33892)	(0.11866)	(0.20640)	(0.34232)	(0.68768)	(0.38612)	(0.93717)

Source: Author’s calculations.

## 6. Conclusion

The empirical findings suggest that the selected stock markets have a long-term dynamic. According to our research, the volatility of the US stock market considerably affected the Indian stock market. We performed a vector error correction model test to assess the stationary conditions of the series. This test demonstrated that the sequence is stationary. However, the variables become constant after considering the original difference. Cointegration tests show that the Indian stock market is integrated over time owing to the presence of a cointegration vector. A model of error correction demonstrates conclusively that variables are causal in the long-term. The stock market in India is impacted by those in the United States, Great Britain, Japan, and Germany. In addition, we applied the pairwise Granger causality test, which reveals the lead-lag connection across the markets, to test for short-term causative and informational linkages among diverse pairs of markets. According to our findings, the Indian stock market is neither fully connected nor completely decoupled from the global market. Nonetheless, the amount of integration implies that portfolio diversification can still result in substantial risk reduction and return maximisation in both the short-term and long-term by diversifying their portfolio during a crisis. In recent years, the returns on major US stock indices have dominated the returns for Indian stocks. Similar results show that the bank-dominated financial sectors of the ASEAN five and China are increasingly integrated ( Caporale *et al*., 2021).

The study is prone to various limitations because it relies on secondary sources of data, which have inherent limitations, such as the total number of trading days during the study period for each country were different. So, the sample was adjusted with the help of EViews software. A quick glance at the study highlights clear-cut investment and portfolio diversification opportunities for international investors. This may help regulators formulate better policies concerning price discovery mechanisms. Moreover, it is possible to extend the present study to include global contagion issues between the sample countries. This could be done using high frequency data.

## Data availability

Figshare: Cointegration and Causality Relationship, https://doi.org/10.6084/m9.figshare.20263803.v2 ( Ali *et al*., 2022).

This project contains the following underlying data:

• CHINA A 50(FTXIN9).xlsx
• DAX.xlsx
• NIKKEI.xlsx
• NSE.xlsx
• S&P 500.xlsx
• CAC 40 (FCHI).xlsx
• DJI.xlsx
• HANG SENG.xlsx
• NASDAQ 100.xlsx
• FTSE.xlsx

Data are available under the terms of the Creative Commons Attribution 4.0 International license (CC-BY 4.0).

## Footnotes

^1^

Menon *et al. *(2009) hypothesize that the Indian equity market have insignificant causal relationship with the US and Japanese equity markets during the crisis period by adopting the “Engle-Granger test” of co-integration. Yarovaya and Lau (2016) also examined the asymmetric causality test results supporting evidence of the coupling and decoupling hypothesis.

^2^
A long-term equilibrium relationship between security margin trading and systemic risk volatility demonstrated by the Johansen co-integration test ( Zhao *et al.*, 2019). Johansen’s cointegration test indicates an average relationship between the Indian and Shanghai stock markets and an especially strong relationship between the Indian and Singapore stock markets. A Granger causality test indicates that margin financing contributes to the volatility of systematic risk in a bear market.

^3^

Mukherjee and Bose (2008) posited cointegration, vector auto-regression, vector error-correction models, and Granger causality and found that the U.S. market leads all Asian markets in terms of information.
