# Supplementary material for: Cointegration and causality relationship of Indian stock market with selected world markets
Source: F1000Res. 2024 Aug 13;11:1241. Originally published 2022 Nov 1. [Version 4] doi: 10.12688/f1000research.123849.4 (PMC11339595; doi:10.12688/f1000research.123849.4)
Supplement: Peer review history

# Reviewer report: Reviewer response for version 4

**DOI:** 10.5256/f1000research.169461.r313924

**Published:** 2024-08-21

**Authors:** Uttam Golder

**Affiliations:** 1 Assistant Professor of Dept. of Finance and Banking, Jashore University of Science and Technology, Jessore District, Khulna Division, Bangladesh

The manuscript looks good and has improved significantly. I believe this version can be finally indexed.

Is the work clearly and accurately presented and does it cite the current literature?

Yes

If applicable, is the statistical analysis and its interpretation appropriate?

Partly

Are all the source data underlying the results available to ensure full reproducibility?

Yes

Is the study design appropriate and is the work technically sound?

Partly

Are the conclusions drawn adequately supported by the results?

Yes

Are sufficient details of methods and analysis provided to allow replication by others?

Partly

Reviewer Expertise:

My research interest is Fintech, Environmental, and Climate Financing. I also have an interest in Growth and Banking related field

I confirm that I have read this submission and believe that I have an appropriate level of expertise to confirm that it is of an acceptable scientific standard.

# Reviewer report: Reviewer response for version 3

**DOI:** 10.5256/f1000research.157421.r217940

**Published:** 2023-11-16

**Authors:** Uttam Golder

**Affiliations:** 1 Assistant Professor of Dept. of Finance and Banking, Jashore University of Science and Technology, Jessore District, Khulna Division, Bangladesh

1. I did not find any error correction term.

 2. Unit root test results are shown only on 1st difference. The level is not reported. So, it is not possible to understand whether the VECM model is applicable or not.

 3. Heteroskedasticity and Serial correlation tests have been done using lag 2. Why? Determining VECM, you used lag 3, so you have to use lag 3 for determining heteroskedasticity and Serial correlation

Is the work clearly and accurately presented and does it cite the current literature?

Yes

If applicable, is the statistical analysis and its interpretation appropriate?

Partly

Are all the source data underlying the results available to ensure full reproducibility?

Yes

Is the study design appropriate and is the work technically sound?

Partly

Are the conclusions drawn adequately supported by the results?

Yes

Are sufficient details of methods and analysis provided to allow replication by others?

Partly

Reviewer Expertise:

My research interest is Fintech, Environmental, and Climate Financing. I also have an interest in Growth and Banking related field

I confirm that I have read this submission and believe that I have an appropriate level of expertise to state that I do not consider it to be of an acceptable scientific standard, for reasons outlined above.

# Reviewer report: Reviewer response for version 2

**DOI:** 10.5256/f1000research.154029.r195568

**Published:** 2023-10-10

**Authors:** Nurul Syuhada Baharuddin, Nur Azwani Mohamad Azmin

**Affiliations:** 1 Faculty of Business and Management, Universiti Teknologi MARA Terengganu, Terengganu, Malaysia

Dear Authors,

 Thank you for the thorough revision of the article. We have reviewed the changes made in response to our previous comments, and we are pleased to see that the authors have addressed all the concerns and suggestions. The revisions have significantly improved the clarity, organization, and overall quality of the article.

 Considering the comprehensive revisions of your manuscript, we are confident in changing our previous status from 'approved with reservation' to 'approved'. We appreciate the authors' dedication to improving the work and commend their efforts. Once again, congratulations on your insightful work.

Is the work clearly and accurately presented and does it cite the current literature?

Yes

If applicable, is the statistical analysis and its interpretation appropriate?

Partly

Are all the source data underlying the results available to ensure full reproducibility?

Yes

Is the study design appropriate and is the work technically sound?

Yes

Are the conclusions drawn adequately supported by the results?

Yes

Are sufficient details of methods and analysis provided to allow replication by others?

Partly

Reviewer Expertise:

NA

We confirm that we have read this submission and believe that we have an appropriate level of expertise to confirm that it is of an acceptable scientific standard.

# Reviewer report: Reviewer response for version 2

**DOI:** 10.5256/f1000research.154029.r195569

**Published:** 2023-08-24

**Authors:** Uttam Golder

**Affiliations:** 1 Assistant Professor of Dept. of Finance and Banking, Jashore University of Science and Technology, Jessore District, Khulna Division, Bangladesh

Thanks to the authors to revise their article. However, in this version, I have some critical comments:

1. The authors used a Vector error correction model. However, I can not find any error correction term. It indicates the speed of adjustment and a negative sign indicates a convergence from the short run to the long run and shows a causal relationship of your explanatory variables with the dependent variable. Please report your ECT term
2. The authors did not show any post-estimation test. The authors must have to show those tests to validate their results. Otherwise, the results are questionable.

So please, report those ECT terms and relevant post-estimation test results. Thank you.

Is the work clearly and accurately presented and does it cite the current literature?

Yes

If applicable, is the statistical analysis and its interpretation appropriate?

Partly

Are all the source data underlying the results available to ensure full reproducibility?

Yes

Is the study design appropriate and is the work technically sound?

Partly

Are the conclusions drawn adequately supported by the results?

Yes

Are sufficient details of methods and analysis provided to allow replication by others?

Partly

Reviewer Expertise:

My research interest is Fintech, Environmental, and Climate Financing. I also have an interest in Growth and Banking related field

I confirm that I have read this submission and believe that I have an appropriate level of expertise to confirm that it is of an acceptable scientific standard, however I have significant reservations, as outlined above.

# Reviewer report: Reviewer response for version 1

**DOI:** 10.5256/f1000research.135998.r180157

**Published:** 2023-07-25

**Authors:** Nurul Syuhada Baharuddin, Nur Azwani Mohamad Azmin

**Affiliations:** 1 Faculty of Business and Management, Universiti Teknologi MARA Terengganu, Terengganu, Malaysia

**Abstract**

 The authors provide a comprehensive explanation of the study. However, the researchers did not specify either the year or the data range that was employed in their study.

**Introduction**

 The problem statement is insufficiently addressed, and the authors include findings from other studies that would be more appropriately highlighted in the Literature Review section. The researcher should emphasize the novel aspects and contributions to existing knowledge of the study.

**Literature Review**

 Many of the writings cited in this column are highly regarded, although literature published before 2010 is generally considered outdated. The citation style and sentence structure in the text does not adhere to the prescribed standards of citation formatting. For instance, the first paragraph's line 19 reads, " *Several studies have identified interconnected financial markets between different economies of the world during the 1996-1997 crisis Yang et al. (2003) by applying (VAR) vector auto-regression*”. Please perform an extensive review and make any necessary changes.

 The researchers do not provide any critical analysis or alternative viewpoints to the current body of work; instead, they simply present the actual findings of previous studies. The current composition would benefit from a better structure by being grouped according to the set of arguments that are being criticized.

**Methods**

 The sample size is enough for the study. However, there are some minor errors, such as incorrect table indications (Table 3 indicates an Augmented Dickey-Fuller test statistic rather than a descriptive statistic -in the methodology section, line 6).  The authors should label the symbols to make the narration more understandable. To make the table more organized and consistent, 4 decimal places are advised.

**Results**

 The flow of the results has been stated simply and precisely. Throughout the discussion of the results, however, there was a lack of support from previous investigations. Only four sources are cited in the study to support the conclusion. It is strongly encouraged to include other sources to back up the findings and continue the conversation.

**Conclusion**

 The authors did not specify whether this finding met the objectives and provided a response to the hypothesis. Nonetheless, the authors provided a conclusive summary of the findings in the conclusion. This section contains well-written descriptions of the study's limitations.

Is the work clearly and accurately presented and does it cite the current literature?

Yes

If applicable, is the statistical analysis and its interpretation appropriate?

Partly

Are all the source data underlying the results available to ensure full reproducibility?

Yes

Is the study design appropriate and is the work technically sound?

Yes

Are the conclusions drawn adequately supported by the results?

Yes

Are sufficient details of methods and analysis provided to allow replication by others?

Partly

Reviewer Expertise:

NA

We confirm that we have read this submission and believe that we have an appropriate level of expertise to confirm that it is of an acceptable scientific standard, however we have significant reservations, as outlined above.

# Reviewer report: Reviewer response for version 1

**DOI:** 10.5256/f1000research.135998.r172401

**Published:** 2023-05-22

**Authors:** Uttam Golder

**Affiliations:** 1 Assistant Professor of Dept. of Finance and Banking, Jashore University of Science and Technology, Jessore District, Khulna Division, Bangladesh

**Review Report on Cointegration and causality relationship of Indian stock market with selected world markets**

 The researcher has identified an interesting topic related to the Cointegration and causality relationship of Indian stock market with selected world markets. Thanks to the authors for their effort. However, from my side, some critical issues in the article have been focused:

**Abstract**

1. Overall the formation of the abstract is correct. However, the authors should indicate the nature and types of the data. Also, they should mention the date range.

**Introduction**

1. The introduction part of the paper is very much simplified. It only provided the overall background of the study, and the problem statement was not fully clear to me. However, it fails to signify it's uniqueness and contribution to the respective fields of knowledge. Also, there is no indication for whom this study is important and in which way it is essential. Besides, what is the study's implication, application, and utility?
2. The authors have said, " *Some authors such as Zhang & Hamori (2021), have also focused on the possible…*". But they only cite two authors of an article. They should cite some more articles.
3. Please include a separate paragraph with the structure of your remaining work in this study.

**Literature review**

1. The authors have said, " *The global financial markets are closely interconnected Muthukumaran et al. (2011) and driven by the emotions of the investors…*" The citation and formation of the sentence are not correct. Please correct it.
2. The authors have said, " *Several studies have identified interconnected financial markets between different economies of the world during the during1996-1997 crisis Yang et al. (2003) by applying VAR (vector auto-regression)*". The citation and formation of the sentence are not correct. Please correct it.
3. The citation style is wrong in several places of your work; for example, you have said " *Some others studies such as Rajwani & Mukherjee (2013) have argued…*". This will be as Rajwani and Mukherjee (2013). This type of mistake is all over the documents. Please check the whole paper, and correct it carefully.
4. As FIGARCH is used for the 1st time in this article, please use its full form.
5. The authors have said, " *Some studies such as Fang et al. (2020) also identified that…*". However, the authors said some studies but mentioned only one article. Why? Please mention some others.
6. I do not understand the meaning of your followings sentence, please rewrite it:
*"Lyócsa & Molnár (2020) found that the autoregressive coefficient was negative during COVID-19 (November 2019 to May 2020) with but the stock market uncertainty and fear of virus highly affected the breath of the autoregressive coefficient amidst the COVID-19 crisis."*
7. The following sentence it too large. Please rewrite it:
  " *Nikkinen & Peltomäki (2020) have focused on investors’ crash worries and utilised data on published newspaper articles and web search volumes to address the complex relationship between information supply and demand connected to investor anxiety and their consequences on realised stock market returns, implying and finding that the media contribute to the efficiency of the stock market by enhancing the transmission of information."*
8. At the end of the literature review section, the laps and gaps of the previous study should be identified, but there is no discussion like that. Moreover, the authors have only explained the previous study's empirical results, but there are no critical arguments against the work done previously.

**Methods**

1. Authors have cited Engle-Granger model. But did not cite the article, rather the citation style is wrongly placed. He wrote: “ *Engle-Granger model is used to measure (Stawiarski, 2021) cointegration between…*”. But (Stawiarski, 2021) should be place at last of the sentence, and also Engle-Granger original paper should be cited where they currently cited (Stawiarski, 2021), on in the place of exactly after the name of Engle-Granger.
2. The authors told that “ *The descriptive statistics for all indexes’ returns are shown in Table 3*.”. But actually it is in Table 1. Correct it.
3. Please do not include the software name and it’s version, it is totally unnecessary.
4. Please exclude the following sentence. It does not make any contribution to your paper:
*"The student version of this software is freely available ( https://eviews.com/download/student11/)."*
5. I can not understand the following sentence. Please rewrite it.
*"As a result, a time series with I (0) is stationary; if I (1), the level is stationary* *and the change is stationary."*
6. When, you write any equation, make a number of it, and also explain the symbols, so rewrite it:
  P=m-k
7. Do not use any informal writing. Authors have said that “It is said that the “ *flap of a butterfly’s wings in Brazil could set off a tornado in Texas” (Lorenz, 2000) **that’s** not true in the Indian context.*” So instead of that’s, use that is.
8. The value of all table should be rewritten. The normal trend is to report three/ four digit after the fraction of a number. So in every table correct it. For example, instead of writing 0.000289, please write 0.000, or 0.0003. Do this in all of your table.
9. Do not use any scientific format of your value, for example instead of using -2.78E-06 (it is scientific format of a number), use -0.00000278. Do this in all cases.
10. In table 3, the authors only provided the unit root test results of 1 ^st^ difference, but where is level results? If the level results show the data have no unit root test, the authors can not perform VECM. So, I have to check the level report. Also, this table is not properly organized. Please see the example from this paper how to report unit root test results: https://doi.org/10.1016%2Fj.heliyon.2023.e14454
11. Where is ECT (error correction term) term? Before validating the results, reviewers should observe it
12. The author also did not report the diagnostic test. It is very much important to validate authors results. Please report serial correlation, heteroskedasticity, normality, J-B test, CUSUM, and CUSUsqrt test to make final comment on the article.
13. The authors should also careful about the formation of the sentence. It should be simple and understandable. Professional proofreader might be required.

**Authors must address the followings**

1. The unit root test results of level form
2. The results of Error correction term
3. Results of serial correlation, heteroskedasticity, normality, J-B test, CUSUM, and CUSUsqrt test

Is the work clearly and accurately presented and does it cite the current literature?

Yes

If applicable, is the statistical analysis and its interpretation appropriate?

Partly

Are all the source data underlying the results available to ensure full reproducibility?

Yes

Is the study design appropriate and is the work technically sound?

Partly

Are the conclusions drawn adequately supported by the results?

Yes

Are sufficient details of methods and analysis provided to allow replication by others?

Partly

Reviewer Expertise:

My research interest is Fintech, Environmental, and Climate Financing. I also have an interest in Growth and Banking related field

I confirm that I have read this submission and believe that I have an appropriate level of expertise to confirm that it is of an acceptable scientific standard, however I have significant reservations, as outlined above.

## References

[1] : Financial progress, inward remittances, and economic growth in Bangladesh: Is the nexus asymmetric?. *Heliyon* .2023;9(3) : 10.1016/j.heliyon.2023.e14454 e14454 10.1016/j.heliyon.2023.e14454 36967937 PMC10031341
